# Systemic Lidocaine Infusion for Acute Pain Management in a Surgical Intensive Care Unit: A Single-Arm Pilot Trial

**DOI:** 10.3390/jcm14134390

**Published:** 2025-06-20

**Authors:** Hina Faisal, Faisal N. Masud, Mahmoud M. Sabawi, Nghi (Andy) Bui, Sara A. Butt, George E. Taffet

**Affiliations:** 1 Department of Surgery, Houston Methodist, Houston, TX 77030, USA; 2 Center for Critical Care, Houston Methodist, Houston, TX 77030, USA; fmasud@houstonmethodist.org; 3 Department of Pharmacy, Houston Methodist, Houston, TX 77030, USA; msabawi@houstonmethodist.org (M.M.S.); nvbui@houstonmethodist.org (N.B.); 4 Center for Health Data Science and Analytics, Houston Methodist, Houston, TX 77030, USA; sbutt@houstonmethodist.org; 5 Department of Medicine, Geriatric Division, Houston Methodist, Houston, TX 77030, USA; gtaffet@houstonmethodist.org

**Keywords:** acute pain, critical care, abdominal surgery, lidocaine, feasibility

## Abstract

**Objectives**: Currently, there are a lack of data on the use of systemic lidocaine infusion in critically ill surgical patients, particularly regarding optimal dosing and monitoring. This study aimed to assess the feasibility of conducting a subsequent full-scale, randomized controlled trial (RCT) on the use of systemic lidocaine infusion in surgical intensive care units (ICUs). **Methods**: A single-center, prospective, single-arm pilot trial was conducted at the surgical intensive care unit (ICU) at Houston Methodist Hospital. The study population included 12 subjects over 18 years old who were admitted to the surgical ICU after open abdominal surgery. A low-dose lidocaine infusion of 10–30 mcg/kg/min within 1 h of ICU admission. **Results**: The feasibility outcomes encompassed recruitment, retention, and withdrawal rates. The study initially screened 18 participants, all of whom were successfully enrolled, resulting in a recruitment rate of 100%. However, 6 participants (33.3%) from the enrolled group were subsequently withdrawn for various reasons, resulting in a retention rate of 12 participants (66.7%). All 12 remaining participants were included in the analysis at the baseline stage. The safety outcomes included adverse events and serum lidocaine levels, with no serious adverse events reported. Dizziness and hypertension were the most frequently reported adverse events in their respective categories, affecting 16.7% of patients each. Four patients (33%) exhibited elevated lidocaine levels exceeding 5 mcg/mL; however, no clinical features of lidocaine toxicity were observed. This study adhered to the CONSORT 2010 extension for pilot and feasibility trials. In accordance with these guidelines, no formal hypothesis testing for efficacy was performed. The exploratory outcomes included a reduction in opioid requirements, as measured by morphine milligram equivalents (MMEs), and pain scores. The median MMEs decreased from 22.6 on postoperative day 0 to 2.5 on day 3. The pain scores decreased by 1.09 units per day (β = −1.09; 95% CI: −1.82 to −0.36; *p* = 0.003); however, the absence of a control group limits the robustness of this observation. **Conclusions**: A large-scale, randomized controlled trial to evaluate the safety and efficacy of systemic lidocaine infusion in the surgical intensive care unit (ICU) seems feasible, with minor adjustments to the eligibility criteria and improved collaboration among nurses, anesthesiologists, and surgeons.

## 1. Introduction

The effective management of acute pain remains a significant challenge in critical care, with opioids still serving as the primary analgesics for up to 90% of postoperative ICU patients [1]. However, opioid use is associated with numerous adverse effects, including delayed recovery, prolonged hospitalization, chronic postsurgical pain, and increased healthcare costs [2,3]. This has prompted a growing interest in multimodal analgesia [4] and opioid-sparing analgesic strategies [5].

One such strategy is the systemic use of lidocaine, a local anesthetic traditionally used for nerve blocks, epidurals, and minor procedures [6]. Intravenous (IV) lidocaine, although off-label, has demonstrated anti-inflammatory, anti-hyperalgesic, and gastrointestinal pro-motility effects that make it a compelling adjunct for perioperative pain management [7]. Clinical evidence [8,9,10] suggests that low-dose lidocaine infusions during major surgeries, such as abdominal [11], orthopedic [12], or thoracic procedures [13], can reduce postoperative pain, opioid requirements, and nausea, thereby promoting faster recovery.

Mechanistically, lidocaine primarily blocks voltage-gated sodium channels, inhibiting action potential propagation in neurons [14]. Other mechanisms of action include the blockade of presynaptic muscarinic and dopamine receptors [15,16], the modulation of glutamate and ion channel protein receptors [17], the inhibition of leukotriene B4 [18], the inhibition of histamine release [19], the inhibition of prostaglandin release [20], and other pro-inflammatory cytokines [21]. Pharmacokinetically, lidocaine is 60–80% protein-bound, extensively metabolized in the liver (mainly via CYP3A4), and renally excreted [22]. Its elimination half-life is approximately 90–120 min, although this may be prolonged in cases of hepatic or cardiac dysfunction [9]. At therapeutic levels, lidocaine is generally safe, but elevated serum concentrations can cause central nervous system and cardiovascular toxicity [9,23,24]. Despite its promising analgesic properties, the use of intravenous (IV) lidocaine in critically ill surgical patients remains underexplored due to their heightened vulnerability to drug-related adverse effects from unstable hemodynamics and organ dysfunction. There is a pressing need for clinical studies to assess the safety and efficacy of systemic lidocaine for acute postoperative pain in this high-risk group. Notably, the non-inferiority trial by Fabian et al. [25] demonstrated that IV lidocaine is as effective as thoracic epidural analgesia (TEA) in controlling postoperative pain after major abdominal surgery, with similar opioid use and no increase in adverse events, suggesting IV lidocaine as a less invasive alternative to TEA [25]. Conversely, the multicenter ALLEGRO trial involving 590 patients undergoing colorectal surgery found that IV lidocaine did not improve postoperative gut recovery, pain, or quality of life compared with placebo, though no harm was observed [26]. Additionally, Xu et al. demonstrated that prolonged IV lidocaine reduced movement-evoked pain and opioid use after hepatectomy, but the clinical impact was limited [27]. These mixed findings highlight the need for further research on the role of IV lidocaine in critically ill surgical populations, who are often excluded from such trials due to concerns about hemodynamic instability and organ dysfunction. Therefore, the primary aim of this single-arm pilot trial was to assess the feasibility of conducting a subsequent randomized controlled trial (RCT) to evaluate the safety and efficacy of systemic lidocaine infusion in surgical ICU patients. Feasibility outcomes included recruitment, retention, and withdrawal rates. A secondary aim was to determine the safety of the use of lidocaine infusion in surgical ICU patients by monitoring adverse events (neurotoxicity, cardiotoxicity, and pulmonary toxicities) and serum lidocaine levels. This study adhered to the CONSORT 2010 extension for pilot and feasibility trials. In line with these guidelines, no formal hypothesis testing for efficacy was performed [28]. An exploratory aim was to estimate the effect of lidocaine infusion on reducing opioid requirements per day, measured in MMEs and pain scores.

## 2. Methods

### 2.1. Study Design and Setting

A prospective single-arm pilot trial without a control or comparator group was conducted at Houston Methodist Hospital to evaluate the use of systemic lidocaine infusion for acute postoperative pain. The Houston Methodist Research Institute Institutional Review Board (IRB) approved this study (IRB approval ID number: PRO00027972; approval date: 27 May 2021; study title: Lidocaine Infusion for Acute Pain) and it was registered at ClinicalTrials.gov (NCT06725485). Our manuscript adheres to the ethical standards of our IRB, and procedures were followed in accordance with the ethical standards of the responsible committee on human experimentation and with the Helsinki Declaration of 1975. All study participants provided written informed consent. This pilot trial design followed the explicit recommendations of a consensus statement on the efficacy and safety of intravenous lidocaine for postoperative pain and recovery [19]. However, in this pilot trial, we used a lower dose of lidocaine infusion (10–30 mcg/kg/min) without the loading dose for the maximum duration of 72 h compared with the recommended dosing and duration of lidocaine infusion in the census statement [19]. The low-dose lidocaine infusion protocol used in this pilot trial is explained in the study intervention sections below.

### 2.2. Patient Populations

The study enrolled 12 adult patients who met the predefined inclusion and exclusion criteria. Eligible participants were over 18 years, admitted to the surgical ICU with severe acute postoperative pain (numerical pain score > 7), and had undergone an open exploratory laparotomy for bowel, gallbladder, or pancreatic surgery. Women of childbearing potential required a negative urine or serum pregnancy test prior to enrollment. Patients were excluded if they had received regional or neuraxial anesthesia, in accordance with consensus guidelines that discourage the concurrent use of systemic lidocaine with other local anesthetic interventions due to the risk of additive systemic toxicity [29]. This risk is heightened in critically ill patients, who may have an impaired hepatic metabolism and altered drug clearance, making them more susceptible to local anesthetic toxicity [29]. Additional exclusion criteria included an allergy to amide local anesthetics, heart failure with ejection fraction < 20%, prior liver transplant, hemodynamic instability requiring two or more vasopressors, severe hepatic dysfunction (Child–Pugh Class C or MELD > 20), recent use of investigational drugs, pregnancy or lactation, chronic opioid dependence, and mechanical ventilation with continuous analgo-sedation. Per the consensus guidelines, patients with a history of chronic pain, regardless of opioid use, were also excluded to maintain a homogeneous study population and focus specifically on acute pain management [29].

Written informed consent was obtained from all participants prior to enrollment.

### 2.3. Sample Size

This study was designed as a single-arm pilot trial without a control or comparator group. Accordingly, no formal sample size calculation was performed for primary outcomes. We aimed to enroll up to 18 participants to achieve a final sample of 12–15, allowing for potential screen failures and withdrawals. A sample size between 10 and 20 participants is generally considered adequate for pilot and feasibility studies that are focused on assessing feasibility outcomes. As an exploratory (pilot) study, it was not powered or structured to make comparative efficacy conclusions [30].

### 2.4. Feasibility Timeline

At our institute, approximately > 1000 patients receive abdominal surgery in a year, and ~30% proceed to the ICU from the surgery. Given this study’s inclusion and exclusion criteria and accounting for the fact that other studies may be ongoing at the time of this study, we anticipated enrolling 1–2 patients every month, with our feasibility timeline goal of recruiting 10 patients in 6 months. We received IRB approval on 27 May 2021. However, our local IRB determined that additional oversight was required, and the primary investigator was to submit a status report or safety report for the first five patients enrolled in this pilot study. Study Subject #1 was recruited and enrolled in the study intervention on 2 July 2021. Study Subject #5 was enrolled on 3 September 2021. By the end of the sixth month, study Subject #11 was enrolled in this pilot study, and the targeted goal of the feasibility timeline was achieved.

### 2.5. Recruitment, Enrollment, and Retention

Patient flow through the research process is illustrated in the flow diagram (see Supplementary Text). A total of 18 participants were initially screened and successfully enrolled in the study, yielding a recruitment rate of 100%. However, 6 participants (33.3%) were subsequently withdrawn due to protocol deviations or clinical findings that emerged after enrollment. Reasons for withdrawal included: (1) intraoperative infiltration of bupivacaine at the surgical wound site by the surgical team, which violated the exclusion criteria; (2) failure to initiate lidocaine infusion as per protocol; (3) post-enrollment disclosure of chronic pain syndrome and pre-existing opioid use; (4) elevated lidocaine levels in the setting of acute kidney injury during surgery; (5) determination that the patient did not undergo an open surgical procedure; and (6) development of tachycardia with hypotension upon ICU admission prior to lidocaine administration. Following these exclusions, the study resulted in a final retention rate of 66.7%, with 12 participants completing the study and included in the analysis. All the retained participants (100%) contributed data to the baseline and subsequent analyses.

### 2.6. Study Interventions

In this study, eligible patients admitted to the surgical ICU received a low-dose continuous intravenous lidocaine infusion initiated within one hour of ICU admission. For patients weighing less than 100 kg, dosing was calculated based on the ideal body weight (IBW). The infusion was initiated at 10 mcg/kg/min (equivalent to 0.6 mg/kg/h) and titrated upward in a stepwise manner—first to 15 mcg/kg/min (0.9 mg/kg/h), then to 20 mcg/kg/min (1.2 mg/kg/h), and 25 mcg/kg/min (1.5 mg/kg/h), as tolerated. The maximum allowable dose was 30 mcg/kg/min (1.8 mg/kg/h). For patients weighing ≥ 100 kg, the infusion rate was capped at 2 mg/min, regardless of the calculated weight-based dosing, with the lower of the two thresholds applied to ensure safety. The therapeutic target of the infusion was to maintain a patient-reported or clinically assessed pain score of less than 3, based on the institution’s validated pain assessment scale. Lidocaine infusion was continued for a maximum duration of 72 h or until ICU discharge, whichever occurred first.

Lidocaine administration and monitoring followed institutional nursing protocols. Vital signs—including blood pressure, heart rate, respiratory rate, and oxygen saturation (SpO_2_)—were monitored and recorded every 15 min during the first hour of infusion, every 30 min for the subsequent two hours, and then hourly thereafter, provided the patient remained hemodynamically stable. In the event of hemodynamic instability—defined as sustained abnormal vital signs or clinical deterioration—the monitoring frequency increased to every 15 min until stabilization was achieved. All measurements were electronically documented in the patient’s medical record.

In addition to routine vital signs, bedside ICU nurses performed focused assessments related to lidocaine toxicity. A standardized neurological assessment was conducted at baseline and then every four hours during the infusion. This included screening for clinical signs, such as seizures, apnea, tinnitus, vertigo, tongue numbness, drowsiness, restlessness, and coma. Nurses also documented lidocaine infusion settings every hour for the duration of the therapy.

To monitor for systemic lidocaine accumulation and minimize the risk of adverse effects, serum lidocaine concentrations were measured at 4, 8, 12, and 16 h after initiation, and then once daily with routine morning laboratory tests. Additional serum level measurements were taken if any symptoms suggestive of lidocaine toxicity were observed. The results were reviewed by the treating physician and/or clinical pharmacist. If the serum lidocaine level exceeded 5 mcg/mL, the infusion was promptly discontinued.

Nursing staff were instructed to immediately stop the infusion and alert the clinical team if any of the following occurred: respiratory rate < 10 breaths per minute; new or worsening confusion, disorientation, or a Richmond Agitation–Sedation Scale (RASS) score less than −2; sustained systolic blood pressure > 180 mmHg or <100 mmHg; heart rate > 110 bpm or <60 bpm; excessive nausea, vomiting, or oral secretions; new or worsening arrhythmias; or lidocaine serum level > 5 mcg/mL.

### 2.7. Outcome Measures

#### 2.7.1. Primary Outcome: Feasibility

The primary outcome of this study was feasibility, assessed through three key metrics: recruitment, retention, and withdrawal rates. These data points reflect the practicality of administering a systemic lidocaine infusion in the ICU setting and are summarized in the participant flow diagram provided in the Supplementary Text.

#### 2.7.2. Secondary Outcome: Safety

The secondary outcome focused on the safety profile of the systemic intravenous lidocaine infusion. Safety was evaluated through the monitoring of adverse events, lidocaine dosing parameters, and serum lidocaine concentrations. Specific adverse events of interest included neurotoxicity, cardiotoxicity, and pulmonary toxicity. Neurotoxicity was defined as the onset of any of the following symptoms within 30 min of starting the lidocaine infusion: blurred vision; tingling or numbness around the mouth (perioral); and generalized tingling, seizures, or coma. Cardiotoxicity was characterized by new or worsening cardiovascular instability occurring within 30 min of infusion initiation. This included sustained hypertension (systolic blood pressure > 180 mmHg), sustained hypotension (systolic blood pressure < 100 mmHg), tachycardia (heart rate > 110 beats per minute), bradycardia (heart rate < 60 beats per minute), or newly developed or increased frequency of arrhythmias. Pulmonary toxicity was defined as new-onset hypoxia (oxygen saturation [SpO_2_] < 88%) or hypercapnia (arterial partial pressure of carbon dioxide [PaCO_2_] > 60 mmHg) in patients with somnolence, confirmed by arterial blood gas analysis within 30 min of infusion initiation. Serum lidocaine levels were measured at scheduled intervals to monitor systemic accumulation and prevent toxicity. A serum concentration > 5 mcg/mL prompted immediate discontinuation of the infusion.

#### 2.7.3. Exploratory Outcomes: Pain Control and Opioid Consumption

Exploratory outcomes included measures of analgesic effectiveness and the opioid-sparing effects of lidocaine: pain scores were assessed using the 11-point Numeric Pain Rating Scale [31], with a target score of <3 indicating adequate pain control. Pain scores were recorded by bedside nurses as part of routine documentation in the electronic medical record. Opioid requirements were calculated as the total daily opioid dose, converted into morphine milligram equivalents (MMEs) using standardized conversion ratios based on the American Pain Society guidelines [32].

Additional Data Collection: Baseline demographic and clinical characteristics were collected for all the participants. These included age, sex, primary diagnosis, relevant past medical history, type of surgical procedure (if applicable), home use of pain medications, reason for ICU admission, requirement for mechanical ventilation at the time of lidocaine infusion initiation, and baseline function of major organs, including hepatic, cardiac, and renal systems.

### 2.8. Statistical Analysis

Baseline characteristics, adverse events, ileus, nausea, vomiting, and return of bowel function were summarized and presented as mean ± standard deviation (SD) or median and interquartile range (IQR) for continuous variables and as number and percentage for categorical variables. To test for differences in the median opioid use per day, as well as median pain scores per day, Skillings–Mack tests were performed. The Skillings–Mack test is a non-parametric statistical test used to analyze data from repeated measures, particularly when missing values exist. It is an extension of the Friedman test, which is traditionally used to detect differences in treatments across multiple test attempts, but the Skillings–Mack test is more flexible and can handle missing data effectively. Mixed effects models were employed to assess the effect of time on opioid use and pain scores. The models included a fixed effect for time (day) and random intercepts for each subject to account for individual variability. This approach allows for the analysis of repeated measures data and accounts for the correlation of measurements within subjects. Additionally, the same mixed effects model was utilized to evaluate the association between lidocaine level and pain scores. Complete case analyses were performed for all the mixed-effect models. All the analyses were performed using STATA version 16 (StataCorp. 2019. Stata Statistical Software: Release 16. College Station, TX, USA: StataCorp LLC).

## 3. Results

Table 1 describes the baseline characteristics from the study data. The study population had a median age of 64.5 years, with an interquartile range (IQR) of 57–66 years, and an equal gender distribution of 50% male and 50% female. The racial composition predominantly comprised White individuals (91.7%). Additionally, 25% of the participants identified as Hispanic or Latino. In terms of medical history, 66.7% of the patients had other diseases, while chronic pain and heart failure with preserved ejection fraction (HFpEF) were each present in 8.3% of the patients. All patients were admitted to the ICU due to a high risk of hemodynamic instability, with 8.3% for acute blood loss. Baseline vital signs indicated a median systolic blood pressure of 141.5 mmHg, a median diastolic blood pressure of 67 mmHg, and a median heart rate of 86 beats per minute. The median oxygen saturation level was 98%. Regarding medication usage, 100% of the patients received acetaminophen (APAP), 91.7% were administered gabapentin, 58.3% received methocarbamol, and 50% were treated with opioids.

Table 2 reports the secondary (safety) outcomes. There were minimal reports of other neurological, cardiovascular, and gastrointestinal events, and no respiratory adverse events were noted. Hypertension was the most common adverse effect affecting three (25%) patients. Overall, the adverse outcomes were infrequent and varied in nature. Five patients (42%) experienced nausea or vomiting.

**Figure 1:**
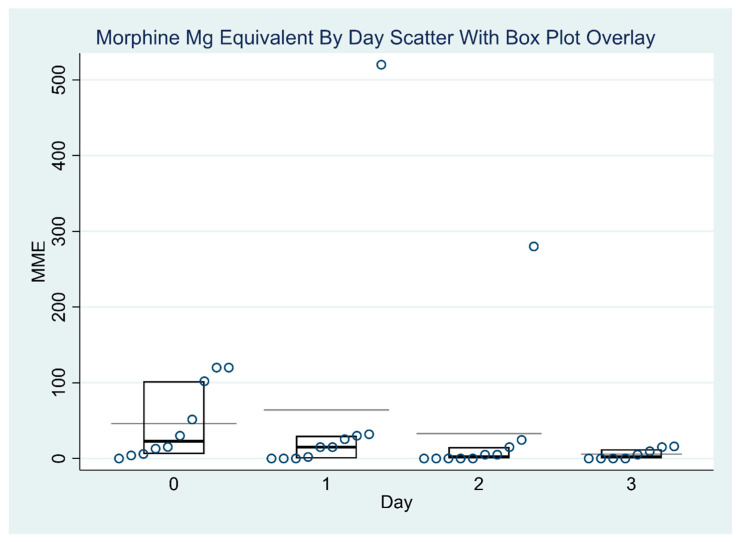
Distribution of opioid requirement in morphine milligram equivalent (MME) by day.

**Figure 2:**
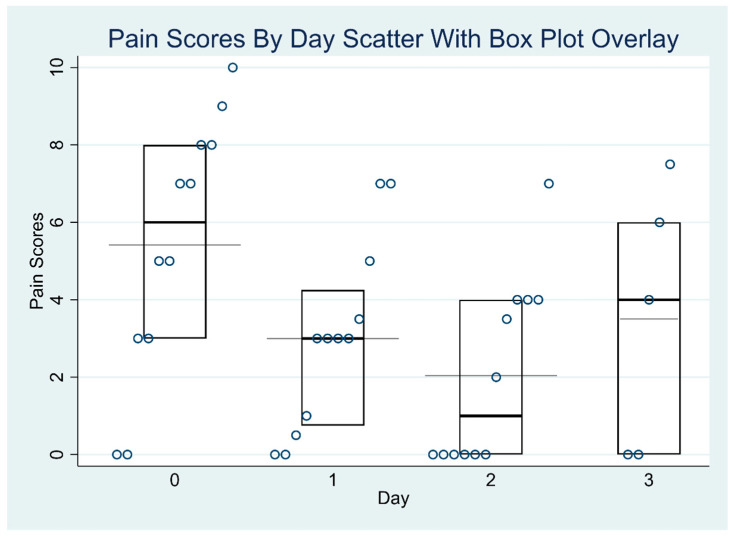
Distribution of pain scores by day.

Table 3 and Table 4 report the exploratory outcomes, including reduction in pain scores and opioid consumption in morphine milligram equivalents (MMEs). Table 3 presents the median pain scores for each day, accompanied by their interquartile ranges (IQRs). Some median values are non-integers (e.g., 0.75, 4.24) because they are interpolated estimates, which can occur when sample sizes are small or when multiple pain scores are tied or evenly distributed. The table also includes the results of the Skillings–Mack test, a non-parametric statistical method suited for analyzing ordinal data with missing values. This test was applied to assess whether there were significant changes in the median pain scores over time. The empirical *p*-value of 0.0250 indicates a statistically significant difference in pain scores across the study days, suggesting that the observed changes are unlikely to be due to chance. Table 4 presents the daily median MMEs values along with the interquartile range (IQR) for each study day. To assess changes in opioid use over time, the Skillings–Mack test was applied. The test statistic was 2.60, with an approximate *p*-value of 0.4584 when ties were not accounted for. Due to the presence of ties in the data, an empirical *p*-value was also calculated using a simulated conditional null distribution, yielding a value of 0.40. Both the approximate and empirical *p*-values exceeded the conventional significance threshold of 0.05, indicating that the observed differences in MME values across the four days were not statistically significant. These results suggest that variations in opioid consumption over time are likely attributable to random variation rather than a systematic trend. Figure 1 and Figure 2 report the distribution of MME values and pain scores over time. There was no significant variation in MME dosages across the four days (*p* = 0.46). The median MMEs decreased from 22.6 on post-operative day 0 to 2.5 on post-operative day 3 (end of the lidocaine infusion). There were significant differences in the pain scores across different days (*p* = 0.025).

Table 5 presents two key analyses using mixed-effects models to examine longitudinal pain scores. First, the model assessing pain scores over time showed the pain score decreased by 1.088 units for each additional day (β = −1.09, SE = 0.37, 95% CI: −1.82 to −0.36, *p* = 0.003), with an average baseline pain score of 4.77. There was substantial variability in baseline pain levels between subjects, and the inclusion of random intercepts was statistically justified (*p* = 0.02). Second, the univariate analysis demonstrated a significant inverse association between lidocaine levels and pain scores; for each one-unit increase in lidocaine, pain scores decreased by approximately 0.31 units (β = −0.31, SE = 0.14, 95% CI: −0.60 to −0.03, *p* = 0.02). The baseline pain score without lidocaine was 3.89, with notable variability across patients. The significant likelihood ratio test (*p* < 0.001) supported the inclusion of random effects, indicating that other unexplained factors may also influence pain variability.

## 4. Discussion

Acute postoperative management in surgical ICU patients is challenging due to hemodynamic instability, organ failure, and adverse effects associated with opioid consumption [33]. The evidence on the safety profile, dose level, and monitoring of systemic lidocaine infusion in the critically ill surgical population is lacking. The goals of this single arm-pilot trial were threefold: (1) to examine the feasibility of conducting a subsequent larger RCT on the use of systemic lidocaine infusion in surgical ICU patients by determining the recruitment, retention, and withdrawal rates; (2) to assess the safety of lidocaine infusion by monitoring signs of toxicities and serum lidocaine levels; and (3) to estimate the effect of lidocaine infusion on pain scores and opioid requirements. Overall, our results indicate that conducting an RCT on systemic lidocaine use for analgesia therapy is feasible, safe, and has the potential to reduce pain scores over time [32].

Systemic lidocaine infusion has been shown to be effective for the management of different types of pain as an adjunct analgesia in a variety of clinical settings [34,35]. Perioperative lidocaine infusion, in doses ranging from 1.5 to 3 mg kg^−1^ h^−1^ (after a bolus of 0 to 1.5 mg/kg), has consistently been shown to improve postoperative pain scores in patients undergoing open or laparoscopic abdominal surgery [31,32,36]. Toxicity from perioperative lidocaine infusion is exceedingly rare [5,37]. If lidocaine toxicities occur, then they tend to occur frequently in older patients with heart failure and significant liver dysfunction [38,39]. Despite its promising analgesic properties, the use of intravenous (IV) lidocaine in critically ill surgical patients remains underexplored. This population presents unique challenges, including altered pharmacokinetics, impaired hepatic clearance, and a higher risk for drug-related adverse effects due to hemodynamic instability and multi-organ dysfunction. As such, there is a pressing need for feasibility and safety data to inform future randomized trials. Several recent studies have investigated the efficacy of IV lidocaine in perioperative settings, though often in more stable, elective surgical populations. Notably, the non-inferiority trial by Fabian et al. [25] demonstrated that IV lidocaine was comparable with TEA for postoperative pain control following major abdominal surgery, with similar opioid consumption and no increase in adverse events—suggesting IV lidocaine as a potentially less invasive alternative to TEA. In contrast, the large, multicenter ALLEGRO trial involving 590 patients undergoing colorectal surgery found no significant benefit of IV lidocaine on postoperative gastrointestinal recovery, pain scores, or quality of life compared with placebo [26]. Another study by Xu et al. [27] reported modest benefits of prolonged IV lidocaine infusion in reducing movement-evoked pain and opioid use after hepatectomy, although the overall clinical impact was limited. In recent years, several systematic reviews of the use of perioperative lidocaine infusion for pain management have been published, including a Cochrane review in which 45 randomized controlled trials with a total of 2802 participants were thoroughly analyzed [35,40]. This provided the foundation and the very first evidence of the potential benefits and safety data of lidocaine infusion for acute pain management but retained significant limitations due to inconsistencies in dosing regimens, the length of administration, and the lack of lidocaine serum monitoring for efficacy and safety profiles that have limited its wide application in clinical practice. In addition, data on ICU patients with organ dysfunctions such as decreased cardiac output, kidney dysfunction, or hepatic impairment were not examined in detail in these studies. Therefore, the present trial evaluated systemic lidocaine at a fixed dose range from 10–30 mcg/kg/min, over a maximum of 72-h duration, and with a lidocaine level monitoring capacity. This standardized regimen helps to address the above limitations, adds knowledge to the current body of evidence on the use of IV lidocaine, and has laid the groundwork for future RCTs. None of the patients experienced serious adverse events during the study period. However, central nervous system adverse events (dizziness) that led to the discontinuation of lidocaine infusion were observed in four patients (33.3%), which included three cases of dizziness and one case of tingling. Four patients experienced cardiovascular events (hypertension or tachycardia. Five patients (42%) experienced nausea or vomiting during lidocaine infusion. The inherent limitations of a pilot design did not allow for a conclusion on whether the adverse events were related to lidocaine infusion. These adverse effects may be related to lidocaine or may be part of the postoperative recovery process.

We also explored the relationship between serum lidocaine levels and infusion doses. Four patients (33.3%) had lidocaine levels exceeding 5 mcg/mL while receiving infusion rates greater than 10 mcg/kg/min. However, six patients (50%) tolerated doses above 10 mcg/kg/min without elevated serum levels. Notably, patients with elevated lidocaine levels did not exhibit any clinical symptoms. In contrast, neurological adverse events, such as vertigo, were observed in patients with lidocaine levels below 5 mcg/mL. Previous studies have reported that serum lidocaine levels typically remain below 5 mcg/mL, even with infusion rates of 25–33 mcg/kg/min. However, in those cases, the levels may not have been measured at a steady state, making interpretation difficult [29]. These findings highlight significant interpatient variability in lidocaine metabolism, underscoring the necessity of individualized dosing strategies. While a serum lidocaine concentration above 5 mcg/mL is traditionally viewed as a cautionary threshold, clinical decisions should prioritize patient symptoms over serum levels alone. Mild neurological symptoms may emerge at concentrations above 5 mcg/mL, with serious toxicity more commonly observed at levels exceeding 6–7 mcg/mL [24]. Our pilot trial findings demonstrate significant interpatient variability in lidocaine metabolism: some patients had serum levels above 5 mcg/mL at infusion rates >10 mcg/kg/min, while others tolerated similar doses without elevated levels. Importantly, clinical symptoms did not consistently correlate with serum concentrations, with neurological adverse events occurring even below 5 mcg/mL. Thus, relying solely on serum lidocaine levels is limited, and clinical symptoms should guide dosing decisions. Overall, these findings highlight the need for a patient-centered approach to lidocaine dosing that balances analgesic efficacy with safety. We recommend individualized dosing with close monitoring, consistent with consensus guidelines advocating a “high-risk” approach [29]. Further research is required to define better safe and effective therapeutic ranges, particularly in critically ill populations with altered pharmacokinetics

Furthermore, we evaluated the relationship between lidocaine levels and pain scores. A univariate analysis found that each 1 mcg/mL increase in lidocaine level is associated with a 0.311 reduction in pain score. The clinical effects of lidocaine in pharmacologic studies were associated with a serum lidocaine range of 1.5–5 mcg/mL [36]. Consistent with the previously published literature [23,35,40], we observed a decrease in pain scores of 1.09 units per day (β = −1.09; 95% CI: −1.82 to −0.36; *p* = 0.003). Additionally, opioid requirements declined markedly from 22.6 MMEs on postoperative day 0 to 2.5 MMEs by postoperative day 3. However, the absence of a control group limits causal inference, and these findings should be interpreted with caution. Although the literature suggests that a 2-point change in pain scores on a 0–10 numeric rating scale may represent a clinically meaningful difference [41], we did not specify a fixed threshold due to the exploratory nature of this study. Given the feasibility design, definitive conclusions cannot be drawn, and we emphasize the need for future randomized controlled trials to further investigate the relationship between lidocaine levels and analgesic effects on pain scores.

This study has several limitations, as well as important implications for clinical practice and future research. First, it is essential to emphasize that this was a feasibility study, and it not designed or powered to formally test hypotheses regarding clinical outcomes. As supported by the existing literature, hypothesis testing in feasibility studies is inappropriate and may lead to misleading conclusions [42]. Therefore, no definitive conclusions can be drawn regarding the safety or efficacy of systemic lidocaine infusion in critically ill surgical patients. The single-center pilot trial design limits the generalizability of our findings, and the inability to blind participants and ICU staff may have introduced bias. Although we observed a reduction in opioid consumption over the three-day postoperative period, this observation should be interpreted cautiously due to the small sample size and instances of missing data, both of which could affect the reliability and external validity of the results. The observed reduction in opioid use (measured in MMEs) may have been influenced by several confounding factors, including natural postoperative recovery, concurrent opioid administration, individual pain tolerance, or the effects of intravenous lidocaine. This study was not designed to distinguish between these potential contributors. Despite these limitations, our findings underscore the need for more comprehensive, hypothesis-driven research. Larger, multicenter studies involving diverse surgical populations and critical care settings are necessary to validate and extend our observations. Future research should also aim to elucidate the pharmacokinetics of lidocaine in critically ill patients and evaluate clinical outcomes to guide evidence-based dosing strategies and safety protocols in the perioperative and intensive care settings.

During the feasibility testing of the lidocaine infusion protocol, we identified several implementation barriers within the ICU setting. A primary challenge was the limited familiarity of ICU staff, particularly nurses and intensivists, with the use of systemic lidocaine in the ICU for the pain management. This lack of experience contributed to hesitancy in initiating infusions, inconsistent monitoring practices, and concerns about potential toxicity. One key issue involved the timing and technique of blood draws for lidocaine serum levels. Occasionally, blood samples were inadvertently drawn from the same intravenous site used for lidocaine infusion, leading to spuriously elevated serum concentrations. This was addressed through targeted nursing education, emphasizing the importance of drawing blood from a separate line or site. Additionally, the timing of serum lidocaine levels was initially inconsistent, with samples obtained either too early or too late relative to the infusion start. To resolve this, we revised the electronic medical record (EMR) lidocaine order set to better align laboratory timing with the expected delivery and administration of the infusion. We also encountered variable levels of acceptance and understanding among anesthesiologists and surgeons. Some clinicians expressed concerns about systemic toxicity or misinterpreted the study’s eligibility criteria, particularly the exclusion of patients receiving regional anesthesia. These observations underscore the importance of clear, multidisciplinary communication during the protocol rollout.

To address these barriers, we propose several practical solutions: (1) structured in-service training for ICU nursing staff focused on lidocaine pharmacology, administration, monitoring, and safety precautions; (2) revisions to EMR order sets to ensure the correct timing of drug delivery and laboratory monitoring; (3) multidisciplinary orientation sessions involving ICU teams, surgeons, and anesthesiologists prior to implementation to clarify eligibility criteria, safety considerations, and workflow integration; (4) protocol review to hold quarterly review meetings to evaluate the performance metrics, discuss feedback, and revise the protocol as needed. In summary, systemic lidocaine infusion doses between 10 and 20 mcg/kg/min with monitoring of lidocaine levels allows for proactive intervention and rate reduction, which is essential in preventing the toxicity associated with drug accumulation in critically ill surgical patients. A large-scale definitive RCT to test the safety and efficacy of systemic lidocaine infusion in surgical ICUs will be feasible with a minor adjustment of the eligibility criteria and better and improved communication with ICU nurses, anesthesiologists, and surgeons.

## 5. Conclusions

This pilot trial was conducted to evaluate the feasibility of our study design and methodology in preparation for a larger, adequately powered randomized controlled trial (RCT). The findings demonstrate that, with only minor adjustments—such as improved protocol education, refined eligibility criteria, and enhanced communication across multidisciplinary teams—the study procedures are both practical and implementable in the ICU setting. These results support the feasibility of conducting a larger-scale RCT with more robust handling of outliers and comprehensive data collection to generate definitive conclusions regarding the safety and analgesic efficacy of systemic lidocaine infusion in critically ill surgical patients.

## Figures and Tables

**Table 1:** Baseline characteristics.

Variables	Statistic
Demographics	
Age in years, median (IQR)	64.5 (57–66)
Gender, n (%)	
Male	6 (50.0)
Female	6 (50.0)
Race, n (%)	
African American	1 (8.3)
White	11 (91.7)
Hispanic Ethnicity, n (%)	
Hispanic or Latino	3 (25.0)
Not Hispanic or Latino	9 (75.0)
BMI (kg/m^2^), median (IQR)	27.9 (26.1–30.6)
Past Medical History, n (%)	
Chronic Pain	1 (8.3)
HFpEF	1 (8.3)
Seizure Disorder	0 (0.0)
ESRD	0 (0.0)
Chronic Kidney Disease	0 (0.0)
Reason For ICU Admission, n (%)	
High Risk of Hemodynamic Instability	12 (100.0)
Other Reason	2 (16.7)
Acute Blood Loss	1 (8.3)
Acute Respiratory Failure	0 (0.0)
Hypotension	0 (0.0)
Acute or High Risk for Kidney Injury	0 (0.0)
Acute Liver Injury	0 (0.0)
Sequential Organ Failure Assessment (SOFA) Score	1.5 (1–3)

IQR = Interquartile range, HFpEF = Heart failure with a preserved ejection fraction.

**Table 2:** Safety outcomes.

Adverse Events	n (%)
Hypertension Dizziness	3 (25) 2 (16.7)
Tachycardia Tingling Seizure	1 (8.3) 1 (8.3) 0 (0.0)
Hypotension	0 (0.0)
Arrhythmia	0 (0.0)
Hypoxia	0 (0.0)

Hypertension: Systolic Blood Pressure (SBP) > 180 mmHg; Tachycardia: Heart Rate (HR) > 15% baseline; Hypotension: SBP < 90 or Mean Arterial Blood Pressure (MAP) < 65 mmHg.

**Table 3:** Pain scores.

Median Pain Scores Per Day with Their Corresponding IQR					
Day		Median Pain Score (IQR)			
Day 0		6 (3–8)			
Day 1		3 (0.75–4.25)			
Day 2		1 (0–4)			
Day 3		4 (0–6)			
**Skillings–Mack Test For Difference in Median Pain Scores**					
**Day**	**N**		**WSumCRank**	**SE**	**WSum/SE**
0	12		13.35	5.39	2.48
1	12		0.37	5.39	0.07
2	12		−9.07	5.39	−1.68
3	5		−4.65	3.87	−1.2

Skillings–Mack Statistic: 7.621. *p*-value (No ties): 0.0545. Empirical *p*-value (Ties): 0.0250. Interquartile range (IQR). Weighted Sum of Centered Ranks (WSumCRank).

**Table 4:** Opioid consumption.

Median Opioid Consumption in MME Per Day					
Day		Median MME (IQR)			
Day 0		22.6 (6:102)			
Day 1		15 (0:30)			
Day 2		2.5 (0:15)			
Day 3		2.5 (0:12.3)			
**Skillings–Mack Test for Difference in Median MME Values Per Day**					
**Day**	**Observations**		**WSumCRank**	**SE**	**WSum/SE**
0	10		3.1	5.29	0.59
1	10		6.29	5.29	1.19
2	10		−4.74	5.29	−0.9
3	8		−4.65	4.9	−0.95

Skillings–Mack Statistic: 2.60. *p*-value (No ties): 0.46. Empirical *p*-value (Ties): 0.40. Morphine Milligram Equivalent (MME). Interquartile range (IQR). Weighted Sum of Centered Ranks (WSumCRank).

**Table 5:** Mixed-effects model to assess the effect on pain scores.

Effect on Pain Scores Over Time					
Fixed Effects					
Parameter	Coefficient (β)	Std. Error (SE)	*p*-Value (*p*)	95% CI Lower	95% CI Upper
Day	−1.088416	0.3711784	0.003	−1.815913	−0.3609201
Intercept	4.774222	0.7415311	0.000	3.320848	6.227596
**Random Effects**					
**Effect**	**Variance**	**Std. Deviation (SD)**	**95% CI Lower**	**95% CI Upper**	
Patient ID	2.50	1.70	0.66	9.47	
Residual	5.51	1.44	3.31	9.18	
**Effect of Lidocaine Level on Pain Scores—Univariate Analysis**					
**Fixed Effects**					
**Parameter**	**Coefficient (β)**	**Std. Error (SE)**	***p*-value (*p*)**	**95% CI Lower**	**95% CI Upper**
Lidocaine Level	−0.3151778	0.1463252	0.031	−0.60197	−0.02838
Intercept	3.892022	0.6230131	0.000	2.6709	5.1131
**Random Effects**					
**Effect**	**Variance**	**Std. Deviation (SD)**	**95% CI Lower**	**95% CI Upper**	
Patient ID	2.5773	1.3666	0.91162	7.2865	
Residual	6.3154	0.84914	4.8523	8.2196	

The median duration of therapy, dose, and lidocaine levels were 49.2 (IQR [37.7–56.5]) hours, 10 (IQR [10–15]) mcg/kg/min, and 2.1 (IRQ [1.3–3.5]) mcg/mL, respectively, with only four instances of supratherapeutic levels and no notable changes in vital signs.

## Data Availability

The original contributions presented in this study are included in the article/Supplementary Material. Further inquiries can be directed to the corresponding author.

## Supplementary Materials

The following supporting information can be downloaded at: https://www.mdpi.com/article/10.3390/jcm14134390/s1.
