# Supplementary figures and images for: Systemic Lidocaine Infusion for Acute Pain Management in a Surgical Intensive Care Unit: A Single-Arm Pilot Trial
Source: J Clin Med. 2025 Jun 20;14(13):4390. doi: 10.3390/jcm14134390 (PMC12249832; doi:10.3390/jcm14134390)

Enrollment Flow Diagram

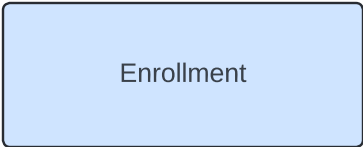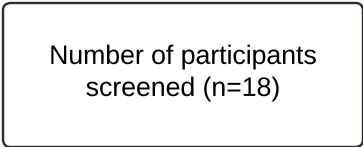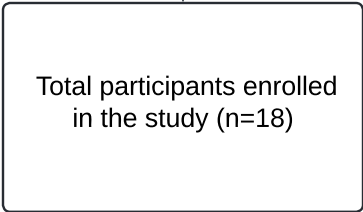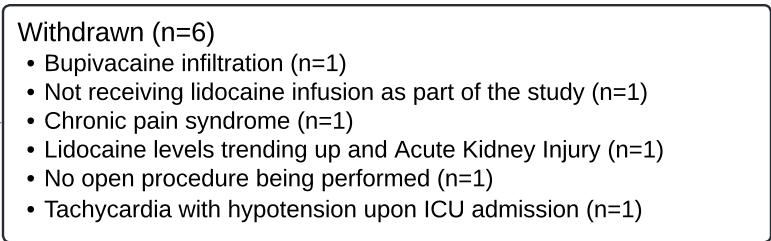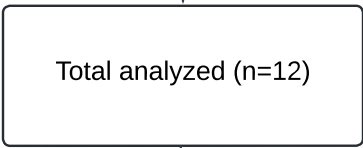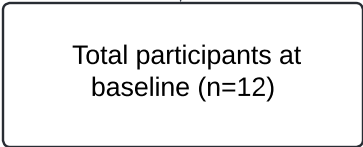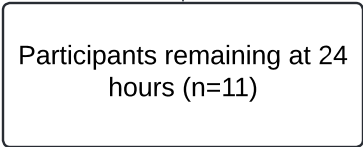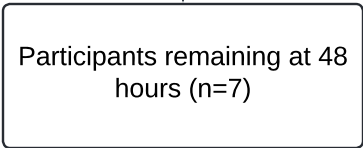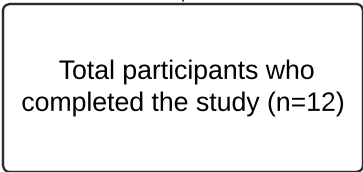

Supplement: Supplementary file 1 [file jcm-14-04390-s001.zip › jcm-3672350-supplementary.pdf]
